# microRNA-378a-3p regulates the progression of hepatocellular carcinoma by regulating PD-L1 and STAT3

**DOI:** 10.1080/21655979.2022.2031408

**Published:** 2022-02-20

**Authors:** Yaqin Li, Tao Zhou, Xianyi Cheng, Dezhi Li, Meng Zhao, Wei V. Zheng

**Affiliations:** a Department of Infectious Disease, Peking University Shenzhen Hospital, Shenzhen, Guangdong, P.R. China; b Intervention and Cell Therapy Center, Peking University Shenzhen Hospital, Shenzhen, Guangdong, P.R. China; c The Second School of Clinical Medicine, Southern Medical University, Guangzhou, Guangdong, China

**Keywords:** Hepatocellular carcinoma, *miR-378a-3p*, PD-L1, immune escape, STAT3

## Abstract

Programmed death ligand 1 (PD-L1) plays an essential role in the development or progression of hepatocellular carcinoma (HCC). MicroRNAs (miRNAs) are small RNA molecules that regulate gene expression during normal and pathophysiological events. Here, we explored the functions and detailed mechanisms of *miR-378a-3p* and PD-L1 in HCC progression. First, *miR-378a-3p* was selected by analyzing miRNA levels in two HCC Gene Expression Omnibus datasets. We found that *miR-378a-3p* levels exhibited a downward trend in HCC and were negatively correlated with PD-L1 levels. Additionally, a dual luciferase assay predicted that *miR-378a-3p* directly targets *PD-L1*. Moreover, the transfection of *miR-378a-3p* mimics into Li-7 and HuH-7 cells effectively decreased the PD-L1 mRNA and protein expression levels, and inhibited Treg differentiation in co-culture models by modulating the expression levels of certain cytokines. Furthermore, the overexpression of *miR-378a-3p* hindered cell proliferation and migration but facilitated apoptosis by repressing STAT3 signaling in HCC cells. In conclusion, *miR-378a-3p* appears to inhibit HCC tumorigenesis by regulating PD-L1 and STAT3 levels. Thus, *miR-378a-3p* may be a potential target for HCC therapy.

## Graphical abstract

**Figure:**
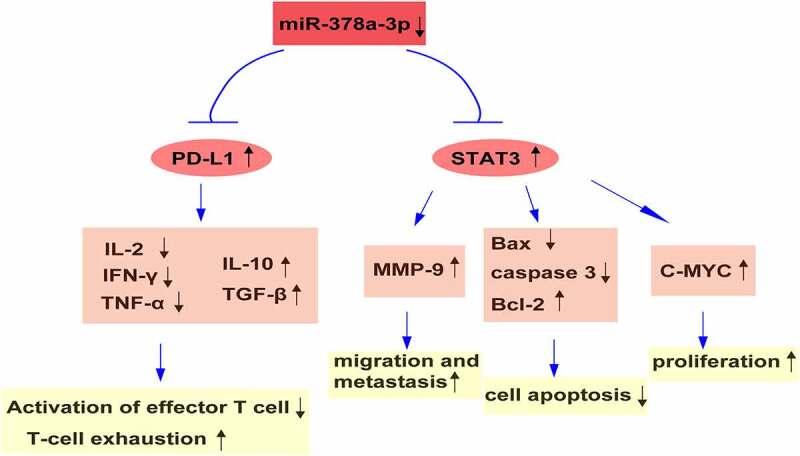

## 1. Introduction

Hepatocellular carcinoma (HCC) is a malignancy exhibiting a high death rate and a year-to-year upward incidence trend globally [1]. Its primary risk factors include co-infection with hepatitis B and C viruses, cirrhosis triggered by excessive alcohol consumption, and nonalcoholic hepatopathy [2–4]. Marked progress in surgical techniques has prolonged survival, but the death and incidence rates of HCC remain equally high due to deficiencies in diagnosis and effective treatment schemes at the early stage. Moreover, surgically resected HCC is prone to relapse [5–7]. Accordingly, the pathogenic mechanism of HCC needs to be understood at the molecular level. This will enable the identification of diagnostic biomarkers and suitable therapeutic targets for the early stage of the disease and facilitate progress in the development of effective treatment schemes.

MicroRNAs (miRNAs) are endogenous non-coding RNAs. They are 22–25 nucleotides [8] and possess the ability to target sequences in the 3’-untranslated region (3’-UTR) of protein-coding genes. This allows them to control gene expression through cleavage and by repressing translation and triggering gross variations in protein content [9,10]. In addition, miRNAs play indispensable regulatory roles in a myriad of cellular processes, such as cell cycle, proliferation, and apoptosis, as well as the growth, progression, and survival of regulatory T cells [11–13]. Furthermore, miRNA expression anomalies have been found in many human cancers [14–16], including HCC [17]. In particular, the expression of *miR-378a-3p*, a tumor-suppressing miRNA, displays anomalies in cervical cancer [18], prostate cancer [19], and breast cancer [20]. Moreover, Qian *et al*. reported that *miR-378a-3p* is a presumptive biological hallmark for HCC diagnosis and prognosis, based on a rational computer-assisted model of biomarker discovery [21]. Nonetheless, the mechanism whereby *miR-378a-3p* influences HCC remains unclear.

Here, our aims were to investigate whether *miR-378a-3p* plays an important role in modulating HCC progression and, if affirmative, to reveal the underlying molecular mechanisms. We explored the roles and possible mechanism of *miR-378a-3p*/programmed death ligand 1 (PD-L1) in the progression of HCC to provide new insights for the clinical diagnosis and treatment of HCC.

## 2. Materials and methods

### 2.1. Tissue sampling

Forty-eight patients with HCC who were not undergoing chemoradiotherapy and were admitted to the Southern Medical University from 2017 to 2019 were selected for the study, and their fresh HCC and non-cancer tissues were collected and frozen. The tissue samples were stored in liquid nitrogen prior to RNA extraction, and experienced pathologists recorded their clinicopathological features. All patients provided written informed consent and the study was approved by the Ethics Committee of the Second School of Clinical Medicine, Southern Medical University. No identifying information has been included in the manuscript.

### 2.2. Cell culture

The cell lines HuH-7 and Li-7 were provided by the American Type Culture Collection (Manassas, VA, USA) and the Cell Bank of the Chinese Academy of Science (Shanghai, China), respectively. Both cell lines were authenticated using short tandem repeat profiling. We used high-glucose (25 mM) Dulbecco’s modified Eagle’s medium (Gibco, Shanghai, China) with 1% penicillin-streptomycin and 10% fetal bovine serum (Sigma, St. Louis, MO, USA) to culture HCC cells, which were stored in a humid incubator (5% CO_2_).

### 2.3. miRNA transfection

GenePharma (Shanghai, China) provided *miR-378a-3p* mimics at different concentrations (10, 20, and 40 pmol) and an miRNA negative control (miR-NC) conjugated with FITC. Detailed sequences are displayed in Table 1. Transfection into Li-7 and HuH-7 cells (1 × 10^6^ cells/mL) was conducted using a Gene Pulser electroporation system (Bio-Rad, Hercules, CA, USA) with 0.2 cm-gap cuvettes at 160 V and a constant time of 12.5 ms. The transfection results were assessed by reverse transcription-quantitative polymerase chain reaction (RT-qPCR).

**Table 1.** Sequences of primers for qRT-PCR and transfection

Name		Sequence
miR-378a-3p	Forward	5’-ACACTCCAGCTGGGACTGGACTTGGAGTCA-3’
	Reverse	5’- CTCAACTGGTGTCGTGGAGTCGGCAATTCAGTTGAGGCC TTCTG-3’
PD-L1	Forward	5’-GTACCTTGGCTTTGCCACAT-3’
	Reverse	5’-CCAACACCACAAGGAGGAGT-3’
GAPDH	Forward	5’-GCACCGTCAAGGCTGAGAAC-3’
	Reverse	5’-TGGTGAAGACGCCAGTGGA-3’
miR-378a-3p mimics	Sense	5’-ACUGGACUUGGAGUCAGAAGGC-3’
	Anti-sense	5’-CUUCUGACUCCAAGUCCAGUUU-3’
miR-378a-3p NC	Sense	5’-UUCUCCGAACGUGUCACGUTT-3’
	Anti-sense	5’-ACGUGACACGUUCGGAGAATT-3’

### 2.4. RT-qPCR

RT-qPCR was performed to measure gene expression levels The TRIzol reagent (GeneAll, Seoul, Republic of Korea) was used for total RNA isolation as specified by the manufacturer’s instructions. The concentration and purity of the obtained RNA were measured using a NanoDrop spectrophotometer (Thermo Fisher Scientific, Shanghai, China) based on the A_260_ and A_280_ values, followed by electrophoresis on 1% agarose gels to assess their integrity.

Complementary DNA (cDNA) was obtained via reverse transcription of total RNA (1 mg) using the ReverTra Ace® qPCR RT kit (cat. no. FSQ‑101; Toyobo Life Science) as specified by the manufacturer’s instructions. Stem-loop primers were used for the synthesis of cDNA from miRNA. Thereafter, the BioFACT 2X Real-Time PCR Master Mix was employed to quantitate target gene expression levels in a StepOnePlus Real-Time PCR System (Thermo Fisher Scientific). The reaction procedure was as follows: 95°C for 3 min, followed by 40 cycles of 95°C for 12s and 60°C for 40s. Gene expression was calculated by the 2^−ΔΔCq^ method [22]. *U6* and GAPDH were used as internal controls, and *miR-378a-3p* expression levels were normalized to *U6* levels, while target gene levels were normalized to GAPDH levels. Genepharma (Shanghai, China) provided all primers, whose detailed sequences are displayed in Table 1.

### 2.5. Dual luciferase experiment

We performed a luciferase reporter assay to probe into the direct *miR-378a-3p-PD-L1* mRNA interplay. In detail, amplification of the *PD-L1* 3’-UTR from HuH-7 cell cDNA was performed using specific primer sequences (1,243 nucleotides) from the 3’-UTR fragments. The 5’ and 3’ ends of the primers were added with appropriate restriction sites to allow cloning into the psi-CHECK-2 vector (Promega, Madison, WI, USA). Subsequently, HEK293T cells were co-transfected with 200 ng of PD-L1 cloning vectors and 40 pmol of *miR-378a-3p* mimics, or with the cloning vectors and a scrambled miRNA (negative control) using the Gene Pulser electroporation system as specified by the manufacturer’s guidelines. After 48 h of culturing, we adopted the Dual-Glo Luciferase Assay System (Promega) to lyse cells for the determination of luciferase activity using a Cytation 5 cell imaging reader (Biotek, Winooski, VY, USA). Firefly luciferase activity (internal control for transfection) was utilized for data normalization.

### 2.6. Western blotting (WB)

As specified by the manufacturer’s protocol, we sequestered cellular proteins using radioimmunoprecipitation assay lysis buffer (Santa Cruz Biotechnology, Dallas, TX, USA) to examine their expression levels. A total of 25 μg of protein per lane was sequestered using sodium dodecyl sulfate-polyacrylamide gel electrophoresis (12%). Subsequently, we adopted the semi-dry blotting method to transfer the proteins onto polyvinylidene fluoride membranes (Roche Diagnostics GmbH, Basel, Switzerland). Then, the membranes were blocked with skim milk powder under shaking for 1 h at room temperature., The membranes were incubated with primary antibodies targeting MMP9 (1/1000, ab76003; Abcam, Shanghai, China), c-MYC (1/1000, ab32072, Abcam), BAX (1/1000, ab32503, Abcam), BCL-2 (1/1000, ab32124, Abcam), cleaved caspase 3 (1/100, ab2302, Abcam), pro-caspase 3 (1/1000, ab32150, Abcam), beta-actin (1/2000, ab8227, Abcam), STAT3 (1/1000, ab68153, Abcam), phospho-STAT3 (pSTAT3, 1/1000, ab76315, Abcam), and PD-L1 (1:2000, sc-293425, Santa Cruz Biotechnology), at 4°C overnight. After rinsing, the membranes were incubated with a moderate amount of secondary antibody (A0208 or A0216; Beyotime, Nantong, China) at room temperature conjugated with horseradish peroxidase. We utilized an electrochemiluminescence system (Roche) for band visualization and a WB imaging system (Sabz Biomedicals, Tehran, Iran) for image capture. Lastly, we used the ImageJ software (National Institutes of Health, Bethesda, MD, USA) for the semi-quantitative assessment of STAT3, pSTAT3, PD-L1, and β-actin (control) protein levels.

### 2.7. 5-ethynyl-2 deoxyuridine (EdU) assay

As specified by the manufacturer’s protocol, Click-iT® EdU Imaging Kits (Invitrogen, Carlsbad, CA, USA) were employed to assess the role of *miR-378a-3p* in HCC cell proliferation. Cells (8 × 10^3^ cells/well) were cultured in 96-well plates and incubated with 10 μL of EdU reagent for 3 h. Subsequently, cells were fixed with 4% formaldehyde for 20 min at room temperature, followed by rinsing with phosphate-buffered saline (PBS). The cells were then incubated in 0.5% Triton X-100 (Sigma, Shanghai, China) at room temperature. Nucleus staining was performed using 1 mL of 4’,6-diamidino-2-phenylindole solution (Sigma); the solution was added to each well and the cells were incubated for 25 min in the dark at room temperature. Then, the staining solution was removed by washing with PBS three times. Finally, pictures of the stained cells were taken, and the cells were quantified under a fluorescence microscope (CKX41-F32FL; Olympus, Beijing, China).

### 2.8. Apoptosis detection

We carried out an annexin V/propidium iodide (PI) assay to evaluate the effect of *miR-378a-3p* mimic transfection on apoptosis. To this end, cells (2.5 × 10^6^) undergoing *miR-378a-3p* mimic transfection were inoculated into six-well plates for 48 h. Subsequently, cells were collected, rinsed with PBS, and stained using an annexin V/PI kit (BD Biosciences, San Jose, CA, USA) as per the manufacturer’s instructions. A MACSQuant10 flow cytometer (Miltenyi Biotec, Bergisch Gladbach, Germany) was used to detect apoptotic cells, and data analysis was conducted using the FlowJo 7.6 software (TreeStar, Ashland, OR, USA).

### 2.9. Wound-healing assay

The impact of *miR-378a-3p* inhibition on HCC cell migration was examined using a wound-healing assay. First, the cells were transfected with *miR-378a-3p* mimics prior to inoculation into 24-well plates (3 × 10^5^ cells/well) and incubated for 24 h. After incubation, a sterile pipette tip was used to scratch the cell monolayer, followed by medium replacement. Images of the cells migrating into the gap area were captured at 0 and 24 h after scratching using an inverted XDS-3 microscope (Optika, Bergamo, Italy). Four fields of view (100x) were selected stochastically, and the cell–cell distance at the invading fronts was measured.

### 2.10. Invasion assays

An invasion assay was used to corroborate the utility of *miR-378a-3p* as a marker of metastatic HCC cell phenotypes. Initially, 8 μm transwells (24-well) were coated with 200 mg/mL Matrigel. HuH-7 or Li-7 cells (0.1 × 10^6^ cells in 100 mL of serum-free medium) transfected with *miR-378a-3p* mimics were added to the upper Matrigel chamber. The lower chamber was filled with RPMI-1640 containing 20% FBS. After 48 h of cell culture, the cells infiltrating the pores were visualized by Giemsa staining, and an inverted microscope was used for cell quantification in five randomly selected fields of view.

### 2.11. Co-culture of HCC and T cells

The peripheral blood mononuclear cells were isolated from the whole blood of healthy donors containing heparin anticoagulant through Ficoll-Hypaque (Lymphodex; Inno-train, Kronberg, Germany) density gradient separation. Subsequently, the cells were centrifuged at 400 × *g* for 30 min, and T cells were isolated by negative immune depletion of CD14^+^, CD15^+^, CD16^+^, CD19^+^, CD34^+^, CD36^+^, CD38^+^, CD56^+^, CD123^+^, and CD235a^+^ (glycophorin A) cells using a magnetic-activated cell sorting kit (Miltenyi Biotec). Next, cells were separated using 5 μg/mL phytohemagglutinin and then stimulated for 24 h prior to joint culture with miRNA-transfected Li-7 and HuH-7 cells. Following 48 h of co-culture at a 1:1 ratio, the suspended T cells were labeled with anti-human CD25 antibodies (302609; BioLegend, San Diego, CA, USA) conjugated with APC following the manufacturer’s instructions. After fixation, permeabilization, and staining with Brilliant Violet 421™-conjugated anti-human FOXP3 (320123; Biolegend), we examined the purity of FOXP3/CD25 Tregs and performed RT-PCR to determine the expression profiles of cytokines, such as TGF-β, IL-10, IFN-γ, TNF-α, and IL-2.

### 2.12. Statistical analysis

Data were analyzed using SPSS 20.0 (Chicago, Illinois, USA) or GraphPad Prism 6.0 (GraphPad, San Diego, CA, USA). All values were displayed as mean ± standard deviation. For data with homogeneous variance in a normal distribution, two-tailed Student’s t-test (two groups) or one-way ANOVA followed by a Bonferroni post hoc test (three or more groups) were used. For data with non-normal distribution or heterogeneous variance, nonparametric Mann–Whitney U (two groups) or Kruskal–Wallis tests followed by a Bonferroni post hoc test were conducted. The correlation between *miR-378a-3p* and *PD-L1* levels was assessed by Pearson correlation analysis. The data presented in Table 2 were analyzed by Fisher’s exact test. Statistical significance was set at *P* < 0.05.

**Table 2.** The relationship of miR‐378a‐3p expression with HCC clinicopathological features

Variables	High miR‐378a‐3p expression (*n* = 21)	Low miR‐378a‐3p expression (*n* = 27)	*p*‐Value
Gender			>0.9999
Female	9	11	
Male	12	16	
Age (years)			0.7715
≤60	10	15	
>60	11	12	
HbsAg			>0.9999
Negative	7	10	
Positive	14	17	
Tumor size (cm)			0.0394
≤5	12	7	
>5	9	20	
Vascular invasion			0.0401
Absent	13	8	
Present	8	19	

Total data from 48 tumor tissues of HCC patients were analyzed. For the expression of miR-378a-3p was assayed by qRT-PCR, the average expression level was used as the cutoff. Data were analyzed by Fisher’s exact test. P-value in bold indicates statistically significant.

## 3. Results

In this study, we aimed to investigate whether *miR-378a-3p* plays an important role in modulating HCC progression and to reveal the underlying molecular mechanisms via PD-L1. Through qRT-PCR detection, we found that *miR-378a-3p* levels exhibited a downward trend in HCC and were negatively correlated with *PD-L1* levels. In vitro experiments indicated *miR-378a-3p-PD-L1* axis regulated the differentiation of CD25+ Foxp3+ Treg cells. Furthermore, the overexpression of *miR-378a-3p* hindered cell proliferation and migration but facilitated apoptosis by repressing *STAT3* signaling in HCC cells. To sum up, it appears that *miR-378a-3p* inhibits HCC tumorigenesis by regulating *PD-L1* and *STAT3*.

### 3.1. miR-378a-3p is expressed at low levels in HCC tissues

**Figure 1.**
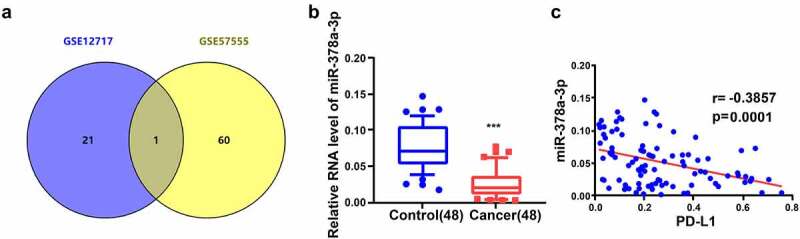
miR-378a-3p is lowly expressed in HCC tissues. (A) HCC GEO datasets (GSE12717 and GSE57555) were analyzed and one lowly expressed gene was identified from the intersection. (B) qRT-PCR analysis of miR-378a-3p expression in 48 pairs of HCC and normal tissues obtained from HCC patients. (C) Negative correlation between miR-378a-3p and PD-L1 expression in HCC samples. ***P < 0.001.

To investigate whether *miR-378a-3p* plays an important role in HCC progression, we first combined and compared the Gene Expression Omnibus (GEO) HCC datasets, GSE12717 and GSE57555, to examine the expression of *miR-378a-3p* in HCC. We found that miR-378a-3p expression was low in HCC (Figure 1(a)). Moreover, RT-qPCR experiments showed that HCC tissues from 48 patients with HCC had lower *miR-378a-3p* expression levels than adjacent non-tumor tissues (Figure 1(b)). Furthermore, the mRNA expression levels of *PD-L1*, an immune checkpoint, displayed an inverse relationship with the expression levels of *miR-378a-3p* in HCC tissues (Figure 1(c)).

Based on the average expression level of *miR-378a-3p*, 48 pairs of tissue specimens fell into high and low *miR-378a-3p* expression groups. In addition, the *miR-378a-3p* expression levels were inversely correlated with tumor size and vascular invasion (*P* < 0.05, Table 2).

### 3.2. PD-L1 is a direct target of miR-378a-3p

**Figure 2.**
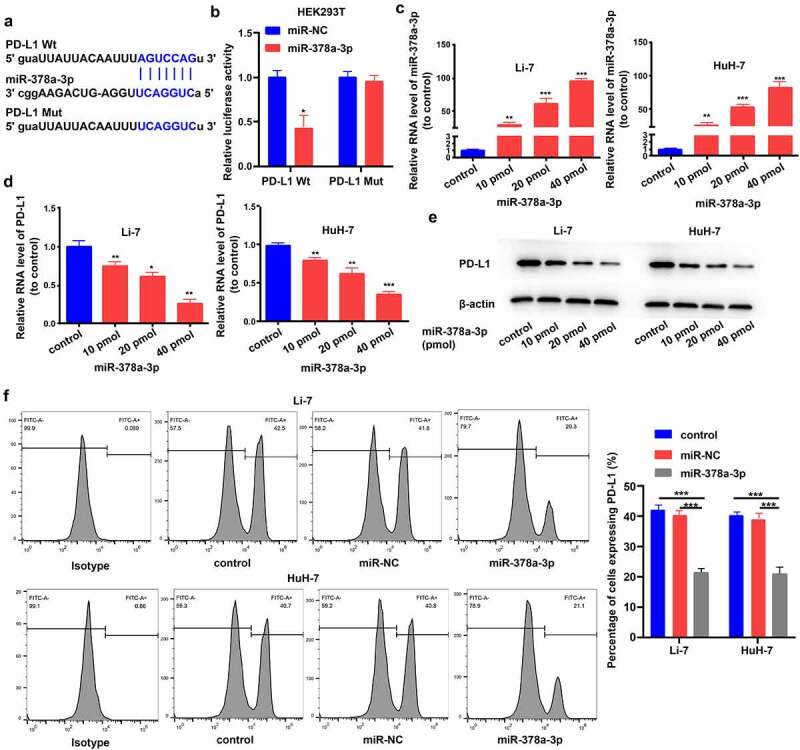
PD-L1 solved as the direct target of miR-378a-3p. (A) Schematic miR-378a-3p putative target sites in 3’UTRs of PD-L1. (B) Dual luciferase reporter assays. (C) The expression level of miR-378a-3p was evaluated using qRT-PCR in HCC cells after transfection. (D) qRT-PCR, (E) Western blot, and (F) flow cytometry were used to evaluate the expression level of PD-L1 after transfection. Data were shown as mean ± SD from three independent experiments. *P < 0.05, **P < 0.01, and ***P < 0.001.

First, we investigated the inhibitory effect of *miR-378a-3p* on the expression of*PD-L1*, via bioinformatics analyses, and predicted binding sites of the miRNA within the 3’-UTR based on data from the starBase (http://starbase.sysu.edu.cn/) and miRDB (http://mirdb.org/) databases (Figure 2(a)). Second, we searched for evidence of the *miR-378a-3p-PD-L1* interaction using a dual luciferase assay. Luciferase activity decreased in HEK293T cells following joint transfection with *miR-378a-3p* mimics and wild-type *PD-L1*; however, luciferase activity was not affected by joint transfection with *miR-378a-3p* mimics and mutant *PD-L1* (Figure 2(b)). This indicated that *miR-378a-3p* directly targets *PD-L1* mRNA.

Then, Li-7 and HuH-7 cells were transfected with *miR-378a-3p* mimics (10, 20, and 40 pmol) to further explore the modulation of *PD-L1* expression by *miR-378a-3p* and the role of *miR-378a-3p* in HCC tumor formation. RT-qPCR analysis showed that transfection with *miR-378a-3p* mimics effectively upregulated *miR-378a-3p* in a dose-dependent manner (Figure 2(c)). Additionally, PD-L1 mRNA and protein levels were compared between cells transfected with miR-NC and those transfected with *miR-378a-3p* mimics, and the results corroborated that *miR-378a-3p* suppresses *PD-L1* expression in HCC cells (Figure 2(d,e)). In subsequent experiments, 40 pmol was taken as the appropriate dose for transfection of miRNA mimics because the strongest effect was seen at this dose. Flow cytometry showed that 40 pmol *miR-378a-3p* markedly decreased PD-L1 expression levels on the cell surface (Figure 2(f)).

### 3.3. miR-378a-3p overexpression reduces HCC cell viability and promotes apoptosis

**Figure 3.**
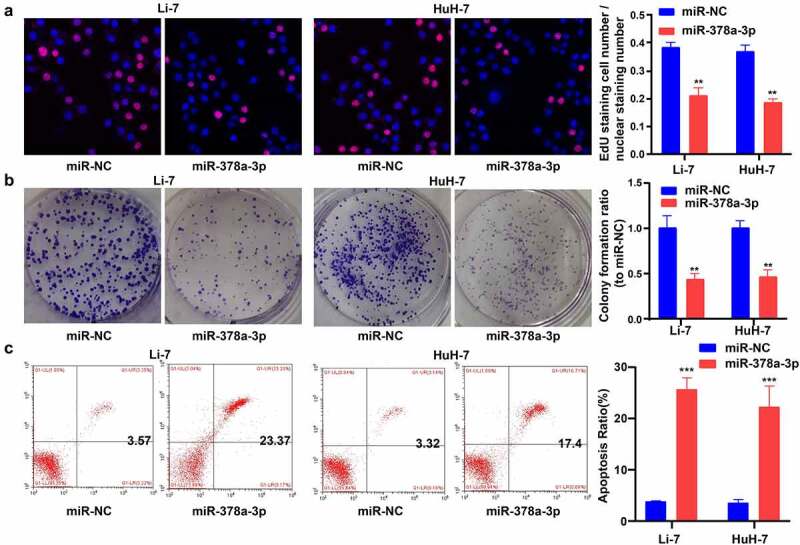
miR-378a-3p overexpression reduced HCC cell viability and promoted cell apoptosis. (A) EdU assay showed that miR-378a-3p reduced HCC cell viability. (B) Colony formation assay showed that miR-378a-3p inhibited HCC cell to form colonies. (C) miR-378a-3p transfection effectively induced apoptosis in HCC cell. Data were shown as mean ± SD from three independent experiments. **P < 0.01, and ***P < 0.001.

**Figure 4.**
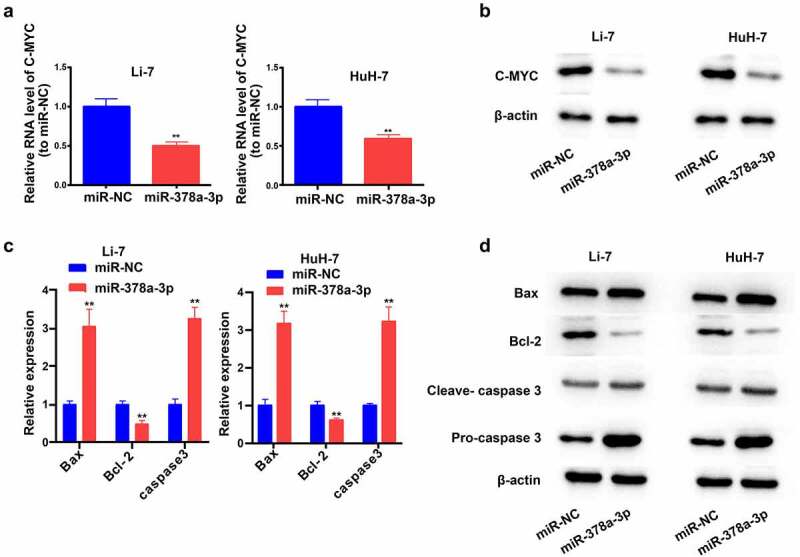
The potential molecular mechanisms of the regulation of miR-378a-3p to the cell viability and apoptosis of HCC cells. (A) C-MYC, as a major gene involving in cell growth, was detected with qRT-PCR. (B) C-MYC was detected with Western blot. (C, D) The levels of major genes related to apoptosis were evaluated using qRT-PCR and Western blot. Data were shown as mean ± SD from three independent experiments. **P < 0.01.

Next, EdU and colony-formation assays were performed to examine the impact of *miR-378a-3p* expression on the growth of HCC cells. Compared with cells transfected with miR-NC, Li-7 and HuH-7 cells transfected with *miR-378a-3p* mimics showed decreased viability (Figure 3(a)). In addition, *miR-378a-3p* mimic transfection negatively affected the colony-forming capacity of Li-7 and HuH-7 cells (Figure 3(b)). Furthermore, the annexin V/PI assay revealed that transfection with the *miR-378a-3p* mimics effectively increased the rate of apoptosis in Li-7 and HuH-7 cells (Figure 3(c)).

c-MYC is an indispensable regulator of cell growth and proliferation. Compared with miR-NC, the *miR-378a-3p* mimics reduced the mRNA and protein levels of c-MYC in Li-7 and HuH-7 cells, suggesting that this may be the mechanism by which *miR-378a-3p* controls HCC cell proliferation (Figure 4(a,b)).

We then assessed the levels of the main genes related to apoptosis pathways (Figure 4(c,d)). WB and RT-qPCR results revealed that the overexpression of *miR-378a-3p* reduced the protein and RNA levels of BCL-2 (a pro-survival gene) in Li-7 and HuH-7 cells, while increasing those of BAX (a pro-apoptotic gene) and caspase 3.

### 3.4. miR-378a-3p impedes HCC cell invasion and migration

**Figure 5.**
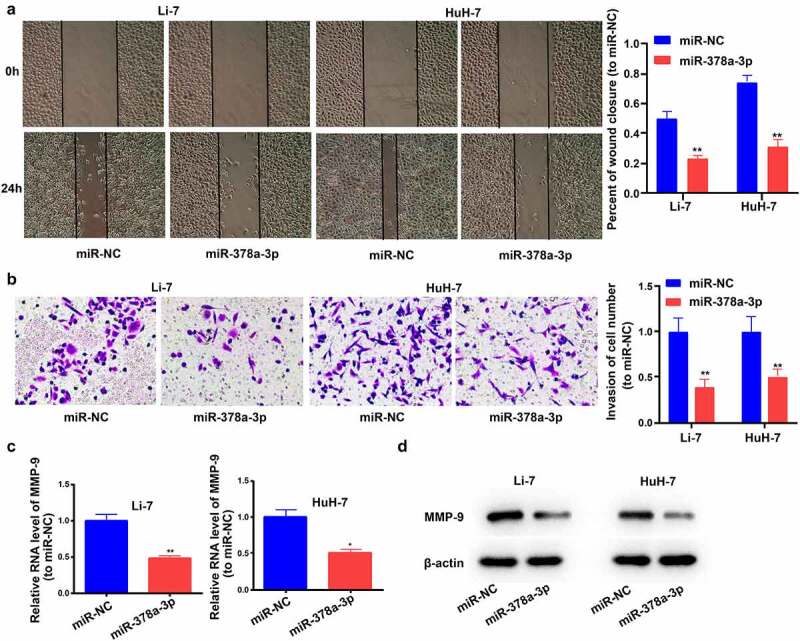
miR-378a-3p overexpression reduced HCC cell invasion and migration. (A) A wound healing assay was performed to investigate cell migration. (B) Transwell assay was employed to investigate cell invasion status. (C) The mRNA level of MMP-9 was detected in HCC cells using qRT-PCR. (D) The protein level of MMP-9 was detected in HCC cells. Data were shown as mean ± SD from three independent experiments. *P < 0.05 and **P < 0.01.

The efficacy of HCC therapy is lower in patients with metastatic HCC. Therefore, we next examined the impact of *miR-378a-3p* on the migration and invasion of Li-7 and HuH-7 cells. Wound-healing experiments showed that the overexpression of *miR-378a-3p* markedly decreased the number of migratory HCC cells in the gap area (Figure 5(a)) and significantly decreased the number of invading Li-7 and HuH-7 cells (Figure 5(b)). Next, we investigated the potential mechanisms by examining the expression levels of MMP-9 (a crucial matrix metalloproteinase in HCC cell metastasis) [23–25]. The transfection of *miR-378a-3p* mimics decreased MMP-9 mRNA and protein expression levels in Li-7 and HuH-7 cells. Collectively, these results suggest that *miR-378a-3p* may have an anti-metastatic effect via the downregulation of MMP-9 expression levels (Figure 5(c,d)).

### 3.5. miR-378a-3p overexpression leads to the downregulation of STAT3

**Figure 6.**
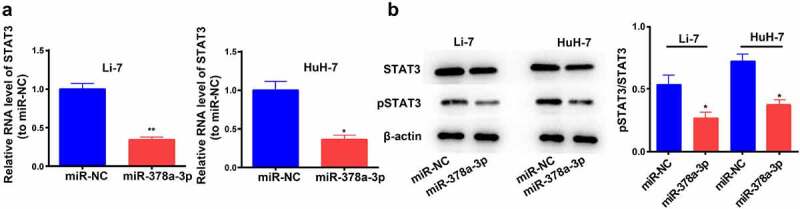
miR-378a-3p overexpression led to down-regulation of STAT3. (A) qRT-PCR and (B) Western blotting were used to evaluate the expression level of STAT3 in HCC cells after transfection. Data were shown as mean ± SD from three independent experiments. *P < 0.05 and **P < 0.01.

STAT3 binds to *miR-378a-3p* in breast cancer [26]. Moreover, several studies have shown that STAT3 exerts a significant impact on the initiation and development of HCC [27–29]. Therefore, we determined its expression levels and found that *miR-378a-3p* overexpression reduced *STAT3* mRNA expression levels (Figure 6(a)). WB analysis showed that *miR-378a-3p* overexpression effectively decreased the protein levels of STAT3 and p-STAT3 in Li-7 and HuH-7 cells, indicating that *miR-378a-3p* probably blocks the formation of HCC tumors by controlling the STAT3 signaling pathway (Figure 6(b)).

### 3.6. miR-378a-3p regulates the differentiation of CD25^+^ Foxp3^+^ Treg cells

**Figure 7.**
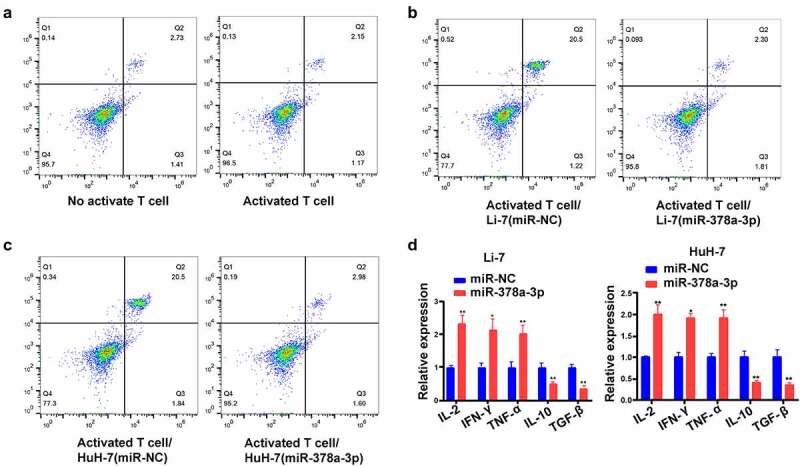
miR-378a-3p modulated the cell differentiation of CD25+ Foxp3+ Treg. (A) The percentage of CD4+CD25+ Foxp3+ Tregs in the CD4+ population was detected using FACS. (B, C) Up-regulation of miR-378a-3p lead to a decrease in Treg induction compared to the negative controls. (D) Detecting cytokine levels with qRT-PCR in co-culture medium. Data were shown as mean ± SD from three independent experiments. *P < 0.05 and **P < 0.01.

Next, HCC/T cell co-culture assays were performed to investigate the involvement of *miR-378a-3p* in Treg development. Following transfection of *miR-378a-3p* mimics, we sequestered T cells from the mononuclear population of peripheral blood. Then, we labeled these cells with antibodies against CD25 cell surface molecules using antibodies against CD25 and Foxp3 conjugated with APC and Brilliant Violet 421™, respectively. There were 2.73% of CD25^+^ Foxp3^+^ Treg cells (most of the inhibitory T cells) in the T cell non-activation group, and 2.15% in the T cell activation group (Figure 7(a)). In addition, the flow cytometry results showed that, unlike with miR-NC transfection, this proportion dropped down following the transfection of *miR-378a-3p* mimics into Li-7 and HuH-7 cells in the co-culture model, suggesting that *miR-378a-3p* probably impedes HCC cell immune escape by suppressing Treg differentiation (Figure 7(b,c)).

Subsequently, we performed RT-qPCR analysis to determine changes in cytokine levels in the co-culture models following transfection of *miR-378a-3p* mimics. We found that *miR-378a-3p* mimics upregulated the *IL-2, TNF-α*, and *IFN-γ* mRNA, while *TGF-β* and *IL-10* were markedly downregulated following *miR-378a-3p* mimic transfection into HCC cells. Thus, it can be concluded that *miR-378a-3p* probably targets *PD-L1* in HCC cells to prevent the differentiation of T cells into the Treg phenotype (Figure 7(d)).

## 4. Discussion

miRNAs are indispensable regulators that are able to negatively regulate target gene expression by inhibiting translation and/or initiating mRNA decay [30]. The regulatory functions of miRNAs in the invasion and proliferation of HCC cells have recently been validated. For example, *miR-612* regulates the invasion of HCC via HADHA-mediated lipid reprogramming [31]. Further, PD-L1 is crucial for the immune escape of HCC [32]. Nonetheless, the role of the miRNA/PD-L1 axis in HCC remains unknown. Thus, the present study aimed to investigate whether the miRNA/PD-L1 axis plays a role in HCC development and immune escape.

PD-L1, also called B7-H1, belongs to the B7 superfamily [33,34]. Immune cells, macrophages, B cells, dendritic cells, and T cells express many members of the B7 family, and their expression levels are increased after activation of the cells [32,35]. PD-L1 also inhibits immune reactions through peripheral T-cell tolerance via interplay with the proteins on the surface of macrophages and T cells. Non-tumor tissues can avoid immunological damage through immunosuppression via the PD-1/PD-L1 pathway [36,37]. However, a myriad of human cancers, including HCC, display anomalies in the regulation of PD-L1 expression [38], which influences tumor formation by maintaining the suppression of adaptive immune reactions and anti-tumor immunity [39–41].

In this study, we integrated two HCC GEO datasets, GSE12717 and GSE57555, to identify pivotal miRNA biomarkers of HCC. *miR-378a-3p* levels displayed a marked decrease in HCC tissues. Using bioinformatics tools, we determined that the *PD-L1* 3’-UTR is a target of miR-378a-3p, which is indicative of its likely function in the immune escape of tumor cells. Subsequently, in vitro experiments showed that *miR-378a-3p* decreased HCC cell viability, invasion, and migration, and promoted apoptosis through the inhibition of STAT3 phosphorylation. Bioinformatics prediction with miRDB (http://mirdb.org/) revealed that TRIM44 is a potential target gene of *miR-378a-3p*. Importantly, TRIM44 is closely related to the phosphorylation of STAT3 [42,43]. Hence, we speculate that *miR-378a-3p* might affect the phosphorylation of STAT3 by regulating TRIM44.

Furthermore, a co-culture model of T cells and HCC cells showed that *miR-378a-3p* may be disadvantageous to Treg induction and HCC immune inhibition, considering the decrease in the number of Tregs in the model after transfection of *miR-378a-3p* mimics. The percentage and activity of Tregs are enhanced in human cancers, and this favors the immune escape of tumor cells by inhibiting the ability of effector T cells to resist tumors [44,45]. Furthermore, previous reports have shown that PD-L1 expression levels are related to the upregulation of Tregs in the tumor environment [46,47]. Concurrently, the involvement of PD-L1 in Treg development and the enhancement of Treg immune-inhibiting capacity has been speculated. Here, we further demonstrated that *miR-378a-3p* mimic transfection into HCC cells in the co-culture model resulted in the downregulation of *IL-10* and *TGF-β* and the upregulation of *IFN-γ, IL-2*, and *TNF-α*, thus reflecting the activity of effector T cells [48,49]. It can be concluded that *miR-378a-3p* has an inhibitory effect on T cell immune escape of HCC cells via PD-L1 decrement. We explored the roles of *miR-378a-3p*/PD-L1 in the progress of HCC and their possible mechanism to provide new insights for the clinical diagnosis and treatment of HCC.

Nevertheless, there were certain limitations to the present study that should be considered. The lack of in vivo evidence is the main limitation, and this will be addressed in future studies. The clinical value of *miR-378a-3p* in HCC also requires further investigation.

## 5. Conclusions

To our knowledge, this is the first study uncovering the role of the miR-378a-3p/PD-L1 axis in HCC. As shown in the graphical abstract, *miR-378a-3p* suppressed PD-L1 expression but promoted a T cell-controlled anticancer response in HCC cells. In addition, *miR-378a-3p* markedly suppressed proliferation, migration, invasion, and other phenotypes in HCC cells by suppressing STAT3 signaling activity and modulating its downstream targets. These results suggest that *miR-378a-3p* hinders HCC progression. Hence, since blocking the PD-L1 pathway is a potential scheme for HCC therapy, *miR-378a-3p* may be a promising novel target for the development of treatments targeting PD-L1-mediated immune response evasion.

## Data Availability

The datasets used and/or analyzed during the current study are available from the corresponding author on reasonable request.

## References

[1] Tang YH, He GL, Huang SZ, et al. The long noncoding RNA AK002107 negatively modulates miR-140-5p and targets TGFBR1 to induce epithelial-mesenchymal transition in hepatocellular carcinoma. Mol Oncol. 2019;13(5):1296–1310. 30943320 10.1002/1878-0261.12487 PMC6487707

[2] Teng CF, Wu HC, Su IJ, et al. Hepatitis B virus Pre-S mutants as biomarkers and targets for the development and recurrence of hepatocellular carcinoma. Viruses. 2020;12(9). DOI:10.3390/v12090945 PMC7552003 32859114

[3] Tseng CH, Hsu YC, Chen TH, et al. Hepatocellular carcinoma incidence with tenofovir versus entecavir in chronic hepatitis B: a systematic review and meta-analysis. Lancet Gastroenterol Hepatol. 2020;5(12):1039–1052. 33007228 10.1016/S2468-1253(20)30249-1

[4] Romano A, Angeli P, Piovesan S, et al. Newly diagnosed hepatocellular carcinoma in patients with advanced hepatitis C treated with DAAs: a prospective population study. J Hepatol. 2018;69(2):345–352. 29551707 10.1016/j.jhep.2018.03.009

[5] Forner A, Reig M, Bruix J. Hepatocellular carcinoma. Lancet. 2018;391(10127):1301–1314. 29307467 10.1016/S0140-6736(18)30010-2

[6] Cho HJ, Kim B, Kim HJ, et al. Liver stiffness measured by MR elastography is a predictor of early HCC recurrence after treatment. Eur Radiol. 2020;30(8):4182–4192. 32189053 10.1007/s00330-020-06792-y

[7] Gao X, Zhan M, Wang L, et al. Timing of DAA initiation after curative treatment and its relationship with the recurrence of HCV-related HCC. J Hepatocell Carcinoma. 2020;7:347–360. 33299823 10.2147/JHC.S279657 PMC7720283

[8] Zhao Y, Wang Z, Zhang W, et al. MicroRNAs play an essential role in autophagy regulation in various disease phenotypes. Biofactors. 2019;45(6):844–856. 31418958 10.1002/biof.1555 PMC6916288

[9] Galagali H, Kim JK. The multifaceted roles of microRNAs in differentiation. Curr Opin Cell Biol. 2020;67:118–140. 33152557 10.1016/j.ceb.2020.08.015 PMC8607530

[10] Wang X, Chen H, Bai J, et al. MicroRNA: an important regulator in acute myeloid leukemia. Cell Biol Int. 2017;41(9):936–945. 28370893 10.1002/cbin.10770

[11] Norouzi M, Yasamineh S, Montazeri M, et al. Recent advances on nanomaterials-based fluorimetric approaches for microRNAs detection. Mater Sci Eng C Mater Biol Appl. 2019;104:110007. 31500008 10.1016/j.msec.2019.110007

[12] Emamgolizadeh Gurt Tapeh B, Mosayyebi B, Samei M, et al. microRNAs involved in T-cell development, selection, activation, and hemostasis. J Cell Physiol. 2020;235(11):8461–8471. 32324267 10.1002/jcp.29689

[13] Emming S, Chirichella M, Monticelli S. MicroRNAs as modulators of T cell functions in cancer. Cancer Lett. 2018;430:172–178. 29800683 10.1016/j.canlet.2018.05.019

[14] Shirjang S, Mansoori B, Asghari S, et al. MicroRNAs in cancer cell death pathways: apoptosis and necroptosis. Free Radic Biol Med. 2019;139:1–15. 31102709 10.1016/j.freeradbiomed.2019.05.017

[15] Staicu CE, Predescu DV, Rusu CM, et al. Role of microRNAs as Clinical Cancer Biomarkers for Ovarian Cancer: a Short Overview. Cells. 2020;9(1). DOI:10.3390/cells9010169 PMC7016727 31936634

[16] Anauate AC, Leal MF, Calcagno DQ, et al. The complex network between MYC oncogene and microRNAs in gastric cancer: an overview. Int J Mol Sci. 2020;21(5). DOI:10.3390/ijms21051782 PMC7084225 32150871

[17] Gupta M, Akhtar J, Sarwat M. MicroRNAs: regulators of immunological reactions in hepatocellular carcinoma. Semin Cell Dev Biol. 2021. DOI:10.1016/j.semcdb.2021.05.025 34049801

[18] Zhang L, Wu ZA. MicroRNA-378a-3p downregulation as a novel biomarker with poor clinical outcomes in cervical cancer. Biomed Environ Sci. 2021;34(3):213–221. 33766217 10.3967/bes2021.026

[19] Mao Y, Li W, Hua B, et al. Circular RNA_PDHX promotes the proliferation and invasion of prostate cancer by sponging MiR-378a-3p. Front Cell Dev Biol. 2020;8:602707. 33634097 10.3389/fcell.2020.602707 PMC7901981

[20] Rong D, Dong Q, Qu H, et al. m(6)A-induced LINC00958 promotes breast cancer tumorigenesis via the miR-378a-3p/YY1 axis. Cell Death Discov. 2021;7(1):27. 33531456 10.1038/s41420-020-00382-z PMC7854648

[21] Qian F, Wang J, Wang Y, et al. MiR-378a-3p as a putative biomarker for hepatocellular carcinoma diagnosis and prognosis: computational screening with experimental validation. Clin Transl Med. 2021;11(2):e307. 33634974 10.1002/ctm2.307 PMC7882078

[22] Livak KJ, Schmittgen TD. Analysis of relative gene expression data using real-time quantitative PCR and the 2(-Delta Delta C(T)) method. Methods. 2001;25(4):402–408. 11846609 10.1006/meth.2001.1262

[23] Thieringer FR, Maass T, Anthon B, et al. Liver-specific overexpression of matrix metalloproteinase 9 (MMP-9) in transgenic mice accelerates development of hepatocellular carcinoma. Mol Carcinog. 2012;51(6):439–448. 21681821 10.1002/mc.20809

[24] Lu L, Zhang Q, Wu K, et al. Hepatitis C virus NS3 protein enhances cancer cell invasion by activating matrix metalloproteinase-9 and cyclooxygenase-2 through ERK/p38/NF-kappaB signal cascade. Cancer Lett. 2015;356(2 Pt B):470–478. 25305454 10.1016/j.canlet.2014.09.027

[25] Ordonez R, Carbajo-Pescador S, Prieto-Dominguez N, et al. Inhibition of matrix metalloproteinase-9 and nuclear factor kappa B contribute to melatonin prevention of motility and invasiveness in HepG2 liver cancer cells. J Pineal Res. 2014;56(1):20–30. 24117795 10.1111/jpi.12092

[26] Yang Q, Zhao S, Shi Z, et al. Chemotherapy-elicited exosomal miR-378a-3p and miR-378d promote breast cancer stemness and chemoresistance via the activation of EZH2/STAT3 signaling. J Exp Clin Cancer Res. 2021;40(1):120. 33823894 10.1186/s13046-021-01901-1 PMC8022546

[27] Huang H, Bu YZ, Zhang XY, et al. LINC01433 promotes hepatocellular carcinoma progression via modulating the miR-1301/STAT3 axis. J Cell Physiol. 2019;234(5):6116–6124. 30317567 10.1002/jcp.27366

[28] Zhu M, Shi X, Gong Z, et al. Cantharidin treatment inhibits hepatocellular carcinoma development by regulating the JAK2/STAT3 and PI3K/Akt pathways in an EphB4-dependent manner. Pharmacol Res. 2020;158:104868. 32407961 10.1016/j.phrs.2020.104868

[29] Wu S, Ye S, Lin X, et al. Small hepatitis B virus surface antigen promotes malignant progression of hepatocellular carcinoma via endoplasmic reticulum stress-induced FGF19/JAK2/STAT3 signaling. Cancer Lett. 2021;499:175–187. 33249195 10.1016/j.canlet.2020.11.032

[30] Fabian MR, Sonenberg N. The mechanics of miRNA-mediated gene silencing: a look under the hood of miRISC. Nat Struct Mol Biol. 2012;19(6):586–593. 22664986 10.1038/nsmb.2296

[31] Liu Y, Lu LL, Wen D, et al. MiR-612 regulates invadopodia of hepatocellular carcinoma by HADHA-mediated lipid reprogramming. J Hematol Oncol. 2020;13(1):12. 32033570 10.1186/s13045-019-0841-3 PMC7006096

[32] Ke MY, Xu T, Fang Y, et al. Liver fibrosis promotes immune escape in hepatocellular carcinoma via GOLM1-mediated PD-L1 upregulation. Cancer Lett. 2021;513:14–25. 33992711 10.1016/j.canlet.2021.05.007

[33] Zhang W, Liu Y, Yan Z, et al. IL-6 promotes PD-L1 expression in monocytes and macrophages by decreasing protein tyrosine phosphatase receptor type O expression in human hepatocellular carcinoma. J Immunother Cancer. 2020;8(1). DOI:10.1136/jitc-2019-000285 PMC7319788 32581055

[34] Arkenau HT, Martin-Liberal J, Calvo E, et al. Ramucirumab plus pembrolizumab in patients with previously treated advanced or metastatic biliary tract cancer: nonrandomized, open-label, phase I trial (JVDF). Oncologist. 2018;23(12):1407–e136. 29853658 10.1634/theoncologist.2018-0044 PMC6292555

[35] Sun TX, Li MY, Zhang ZH, et al. Usnic acid suppresses cervical cancer cell proliferation by inhibiting PD-L1 expression and enhancing T-lymphocyte tumor-killing activity. Phytother Res. 2021. DOI:10.1002/ptr.7103 33970512

[36] Verdura S, Cuyas E, Cortada E, et al. Resveratrol targets PD-L1 glycosylation and dimerization to enhance antitumor T-cell immunity. Aging (Albany NY). 2020;12(1):8–34. 31901900 10.18632/aging.102646 PMC6977679

[37] Zhang YF, Zhang ZH, Li MY, et al. Britannin stabilizes T cell activity and inhibits proliferation and angiogenesis by targeting PD-L1 via abrogation of the crosstalk between Myc and HIF-1alpha in cancer. Phytomedicine. 2021;81:153425. 33310309 10.1016/j.phymed.2020.153425

[38] Pena-Asensio J, Calvo H, Torralba M, et al. Anti-PD-1/PD-L1 based combination immunotherapy to boost antigen-specific CD8(+) T cell response in hepatocellular carcinoma. Cancers (Basel). 2021;13(8). DOI:10.3390/cancers13081922 PMC8073815 33923463

[39] Sasaki S, Nishikawa J, Sakai K, et al. EBV-associated gastric cancer evades T-cell immunity by PD-1/PD-L1 interactions. Gastric Cancer. 2019;22(3):486–496. 30264329 10.1007/s10120-018-0880-4

[40] Cortellini A, Buti S, Bersanelli M, et al. Evaluating the role of FAMIly history of cancer and diagnosis of multiple neoplasms in cancer patients receiving PD-1/PD-L1 checkpoint inhibitors: the multicenter FAMI-L1 study. Oncoimmunology. 2020;9(1):1710389. 32002308 10.1080/2162402X.2019.1710389 PMC6959456

[41] Komura N, Mabuchi S, Shimura K, et al. The role of myeloid-derived suppressor cells in increasing cancer stem-like cells and promoting PD-L1 expression in epithelial ovarian cancer. Cancer Immunol Immunother. 2020;69(12):2477–2499. 32561967 10.1007/s00262-020-02628-2 PMC11027471

[42] Chen L, Yi C, Li W, et al. Inhibition of SPATS2 suppresses proliferation and invasion of hepatocellular carcinoma cells through TRIM44-STAT3 signaling pathway. J Cancer. 2021;12(1):89–98. 33391405 10.7150/jca.47526 PMC7738826

[43] Xiong D, Jin C, Ye X, et al. TRIM44 promotes human esophageal cancer progression via the AKT/mTOR pathway. Cancer Sci. 2018;109(10):3080–3092. 30098109 10.1111/cas.13762 PMC6172051

[44] Whiteside TL. What are regulatory T cells (Treg) regulating in cancer and why? Semin Cancer Biol. 2012;22(4):327–334. 22465232 10.1016/j.semcancer.2012.03.004 PMC3385925

[45] Jie HB, Gildener-Leapman N, Li J, et al. Intratumoral regulatory T cells upregulate immunosuppressive molecules in head and neck cancer patients. Br J Cancer. 2013;109(10):2629–2635. 24169351 10.1038/bjc.2013.645 PMC3833228

[46] Schuler PJ, Schilling B, Harasymczuk M, et al. Phenotypic and functional characteristics of CD4+ CD39+ FOXP3+ and CD4+ CD39+ FOXP3neg T-cell subsets in cancer patients. Eur J Immunol. 2012;42(7):1876–1885. 22585562 10.1002/eji.201142347 PMC3689271

[47] Tauriello DVF, Palomo-Ponce S, Stork D, et al. TGFbeta drives immune evasion in genetically reconstituted colon cancer metastasis. Nature. 2018;554(7693):538–543. 29443964 10.1038/nature25492

[48] Galeano Nino JL, Tay SS, Tearle JLE, et al. The Lifeact-EGFP mouse is a translationally controlled fluorescent reporter of T cell activation. J Cell Sci. 2020;133(5). DOI:10.1242/jcs.238014 32041902

[49] Snyder LD, Chan C, Kwon D, et al. Polyfunctional T-cell signatures to predict protection from cytomegalovirus after lung transplantation. Am J Respir Crit Care Med. 2016;193(1):78–85. 26372850 10.1164/rccm.201504-0733OC PMC4731614

